# Falls, non-accidental falls and syncope in community-dwelling adults aged 50 years and older: Implications for cardiovascular assessment

**DOI:** 10.1371/journal.pone.0180997

**Published:** 2017-07-21

**Authors:** Jaspreet Bhangu, Bellinda L. King-Kallimanis, Orna A. Donoghue, Laura Carroll, Rose Anne Kenny

**Affiliations:** The Irish Longitudinal Study on Ageing, Trinity College, Dublin, Ireland; Cardiff University, UNITED KINGDOM

## Abstract

**Objectives:**

To calculate the prevalence of all falls, non-accidental falls and syncope in an older population and characterize cardiovascular risk profiles.

**Design:**

Prospective, longitudinal cohort study.

**Setting:**

The first two waves of data from the Irish Longitudinal Study on Ageing (TILDA).

**Participants:**

8172 community-dwelling adults aged 50 years and older resident in the Republic of Ireland

**Measurements:**

Self-reported history of all falls, non-accidental falls and syncope in the year preceding the first two waves of data collection. Demographic factors and self-reported cardiovascular conditions were used to characterize cardiovascular risk profiles.

**Results:**

The prevalence of all falls in the past year was 19.2% or 192 per thousand persons and increased with age (50–64 years 17.5%; 65–74 years 19.4%; 75+ years 24.4%). Non-accidental falls had an estimated prevalence of 5.1% or 51 falls per thousand persons and accounted for 26.5% of all falls reported and also increased with age (50–64 years 4.0%; 65–74 years 5.5%; 75+ years 8.0%). The prevalence for syncope was estimated to be 4.4% or 44per thousand persons but did not show a similar age gradient. Participants with at least 5 cardiovascular conditions were more likely to report all falls (OR = 2.07, 95% CI 1.18–3.64, p<0.05) and NAF (OR = 2.89, 95%CI 1.28–6.52, p<0.05).

**Conclusions:**

The prevalence of all falls and non-accidental falls increases with age but the same pattern was not consistently observed for syncope. There is an increased odds of reporting all three outcomes with increasing number of self-reported cardiovascular conditions. Further work is needed to uncover the interplay between cardiovascular disease and subsequent falls.

## Introduction

Falls and syncope are common in older adults. Currently, falls account for 4% of the healthcare budget while syncope accounts for 1–2% of all emergency department (ED) presentations per year in the United States [1, 2]. Given projected changes in global demographics and advancing age, the management of falls and syncope and their consequences will become even more pertinent in the near future[3].

Falls frequently occur due to the environment or accidental events such as trips or slips [4]. These accidental falls become more common with advancing age and are often due to age and disease associated reductions in physical, sensory and cognitive function which make an individual more susceptible to environmental hazards[4].

However, some falls are not accidental but rather are due to drops in blood pressure which may lead to either balance instability or, in some cases, loss of consciousness i.e. syncope[5]. Similar to syncope, non-accidental falls (NAF) have been linked to cardiovascular disorders with a possible common causal pathway and overlap[6]. Dynamic changes in blood pressure with higher rates of orthostatic hypotension have been associated with an increased risk of NAF and injurious falls in community-dwelling adults [7]. Disorders which are known to cause syncope in the elderly including vaso-vagal syncope and carotid sinus syndrome occur in up to 25% of NAF [8, 9] [10–12]. In addition to shared cardiovascular associations there is a higher reported prevalence of depression in patients with both NAF and syncope[13, 14]. Despite the overlap, falls, NAF and syncope are generally reported separately, therefore studies which distinguish between them and examine them in more depth are required [15].

We hypothesise that the prevalence of all falls, NAF and syncope increase with age and share common demographic and cardiovascular risk factors. In order to show this, we calculated the prevalence of all reported falls, NAF and syncope in a population study of community-dwelling adults aged 50 years and over and examined the demographic and cardiovascular health variables associated with all falls, NAF and syncope.

## Methods

### Sample

This study utilised the first two waves of data from The Irish Longitudinal Study on Ageing (TILDA). TILDA is a prospective cohort study of the social, economic and health circumstances of community-dwelling adults aged 50 years and older resident in the Republic of Ireland. The sampling procedure and the study design have been described in detail previously [16, 17]. Briefly, the sampling frame was the Irish Geodirectory, a listing of all residential addresses in the Republic of Ireland. The RANSAM sampling system was used to randomly target households and all household residents aged 50 years or older and their spouse/partner (of any age) were invited to participate in the study. All participants provided written informed consent and ethical approval for the study was granted by the Research Ethics Committee of Trinity College Dublin. All experimental procedures adhered to the Declaration of Helsinki.

The household response rate was 62%, leading to a final wave one sample of 8,172 adults aged 50 and older who completed an in-home interview between October 2009 and February 2011. Follow-up data for wave two were collected between March 2012 and March 2013 (2 year gap between waves). Attrition accounted for a 12% sample reduction and death a further 2.5%.

Data collection involved an in-home computer-aided personal interview conducted by trained social interviewers, a self-completion questionnaire completed and returned by participants in their own time; and a comprehensive health assessment carried out by research nurses in a dedicated health centre or if required, a modified assessment carried out in the participant’s own home. The response rates for the self-completion questionnaire and the health assessment were 85% and 72%, therefore to maximise the sample size, this analysis uses data obtained during the in-home interview only.

### Measures

Syncope—Participants were asked if they had experienced a faint or blackout in the past twelve months (yes/no). Syncope was defined as at least one syncopal event in the past year.

All falls—Participants were asked if they had fallen in the past year (yes/no). A fall was defined as at least one reported fall in the last year.

Non-Accidental Falls (NAF)—Participants were asked if any of the falls they had experienced in the last year were non-accidental, i.e. with no apparent or obvious reason (yes/no). A NAF was defined as at least one reported NAF in the last year.

Demographic and Health Status variables—In addition to demographic variables (age and sex), participants were asked to self-report any doctor diagnosed cardiovascular conditions including: hypertension, angina, a heart attack, congestive heart failure, diabetes or high blood sugar, a stroke (cerebral vascular disease), mini-stroke or transient ischaemic attack, high cholesterol, a heart murmur, an abnormal heart rhythm (arrhythmia). Due to small numbers within individual variables, and the potential for overlap, a composite cardiovascular disease exposure variable was generated. This indicator variable for cardiovascular conditions was created by summing the number of conditions.

All medications taken regularly were coded using the World Health Organisation Anatomical Therapeutic Chemical (ATC) Classification system[18]. Anti-hypertensives were identified by ATC codes beginning with C02, C03, C07, C08 or C09. Depressive symptoms were assessed using the 20-item Centre for Epidemiological Studies Depression scale (CES-D)[19] where scores of < 16 indicated insignificant symptoms for depression; >16 and < 26 indicated moderate to severe depressive symptoms and > 26 indicated severe depressive symptoms. All demographic and health variables were obtained at wave one. Unsteadiness during walking was divided into a binary variable based on self-reported steadiness while walking [20].

### Statistical analysis

Prevalence estimates were weighted with respect to age, sex and education to the Quarterly National Household Survey (2010) to ensure that data were nationally representative. Incidence at wave 2 was calculated using the sub-sample who did not report a syncopal event or fall in wave 1. An attrition weight was used to adjust for loss to follow-up through participant refusal, loss of contact or death between waves. Cross tabulation was used to estimate prevalence and 95% confidence intervals. Inferential statistics (design-based *F* statistic, *p* <0.01) were computed for age and sex estimates using analysis of variance.

To better understand the relationships between basic demographic variables and general health with respect to all falls, NAF and syncope, logistic regression was used, without survey weights with univariate odds ratios calculated. Multivariate logistic regression models were built using causal modelling including age, sex, depressive symptoms, anti-hypertensive drugs, self-reported cardiovascular conditions, and self- reported unsteadiness while walking. A second model was built analysing CVD as a composite number of conditions. Odds ratios, *p*-values and 95% confidence intervals were used to assess the association with potential risk factors. A *p*-value <0.05 represented statistical significance. All analyses were conducted using Stata 12.1 Statacorp LP.

## Results

The total number of participants aged 50 years and over included in the study was 8,172 (50-65years (n = 4664; 57%), 65–74 years (n = 2159; 26.4%), 75+ years (n = 1352; 16.5%)); mean age 63.7 years (SD 9.7); 55.6% (n = 4,724) female.

### Prevalence of all falls, NAF and syncope in the Irish population

Baseline descriptives for groups reporting all falls, NAF and syncope are provided in Table 1.

**Table 1:** Baseline variables for all participants reporting all falls (n = 1,579), non-accidental falls (NAF)(n = 406) and syncope (n = 363) in wave one of TILDA.

Variable	Falls N (%)	NAF N (%)	Syncope N(%)
Age (years)			
50–65	882 (56)	234 (58)	186 (51)
65–75	433 (27)	105 (26)	107 (29)
75+	264 (17)	67 (17)	70 (19)
Gender (female)	919 (58)	238 (59)	177 (49)
Anti-hypertensive medications^1^	634 (40)	198 (49)	169 (47)
Unsteadiness^2^	311 (23)	132 (46)	72 (24)
Number of cardiovascular conditions^3^			
0	346 (22)	70 (17)	54 (15)
1	901 (57)	236 (58)	213 (59)
2	209 (13)	63 (16)	51 (14)
3	74 (5)	15 (4)	25(7)
>3	49 (3)	22 (5)	20 (6)
Depression^4^			
None/insignificant	1006 (64)	212 (52)	197 (54)
Moderate	323 (20)	106 (26)	82 (23)
Severe	219 (14)	78 (19)	75 (21)

^1^As coded by the WHO Anatomic Therapeutic Chemical (ATC) Classification System; anti-hypertensive medication with ATC unsteadiness during walking code C02, C03, C07, C08, C09

^2^Self reported unsteadiness during walking

^3^Self- reported doctor diagnosed cardiovascular conditions including angina, hypertension, congestive cardiac failure, diabetes, stroke, transient ischemic attack, high cholesterol, cardiac murmurs and cardiac arrhythmia

^4^ As measured by Centre for Epidemiological Studies scale (CES-D) scale; scores of < 16 indicated insignificant symptoms for depression; >16 and < 26 indicated moderate to severe depressive symptoms and > 26 indicated severe depressive symptoms.

**Fig 1:**
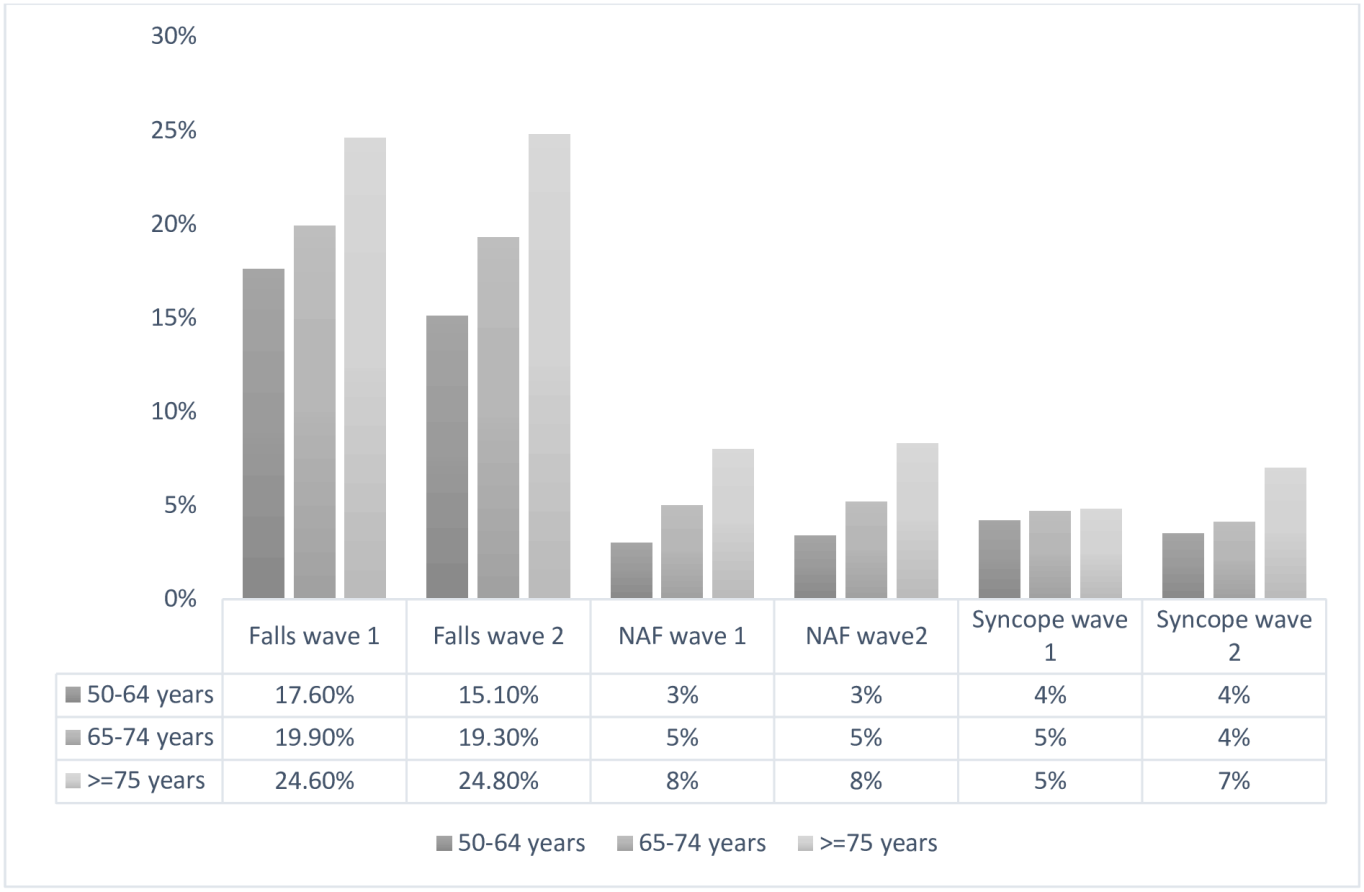
Prevalence and incidence of all falls, non-accidental falls (NAF) and syncope. Prevalence (wave one) and incidence (wave two) based on self-reported data from TILDA participants (n = 8172).

The prevalence (wave 1) and incidence (wave 2) of all falls, NAF and syncope stratified by age is presented in Fig 1. The overall prevalence of all falls in the past year was 19.2% or 192 per thousand persons and increased with age (50–64 years 17.5%; 65–74 years 19.4%; 75+ years 24.4%) (F(2,1240.7) = 15.92, p<0.001). Falls were more prevalent in females (20.1%) compared to males (18.2%).

NAF had an estimated prevalence of 5.1% or 51 falls per thousand persons and accounted for 26.5% of all falls reported. Again, the prevalence increased with age (50–64 years 4.0%; 65–74 years 5.5%; 75+ years 8.0%) (F(2,1247.3) = 15.15, p<0.001).

The prevalence for syncope was estimated to be 4.4% or 44 per thousand persons (Fig 1). Prevalence was similar for males (4.4%) and females (4.5%) and did not differ when stratified by age in wave one (F(2,1235.8) = 0.87, p< 0.10) (Fig 1).

### Incidence

The estimated overall incidence of all falls was 17.5% while NAF was 5%. In both cases, incidence was highest for those aged 75 years and older (all falls 24.8%; NAF 8%) (Fig 1). Overall, the estimated incidence of syncope was 4.2%. There was an age-related increase in incidence, with those over 75 years demonstrating a higher incidence of syncope (50–64 years 3.5%; 65–74 years 4.1%; 75+ years 7%) (F(2,1228.8) = 12.56, p <0.05). The estimated incidence for males aged 75 and older was 5.2% compared to 8.3% in females of the same age.

### Clinical characteristics

Tables 2 and 3 illustrate the univariate and multivariate associations between cardiovascular conditions with all falls, NAF and syncope. In univariate analysis, cardiovascular conditions including angina, heart failure, stroke, TIA, diabetes and arrhythmia displayed an association with all three outcomes. When adjusted for potential confounders none of the cardiovascular conditions showed an individual association with all three outcomes. Heart murmur showed an association with all falls 1.35(95% CI 1.07–1.71, p<0.05) and syncope 1.71 (96% CI 1.17–2.52, p<0.05); self reported arrhythmia was associated with all falls 1.30 (95% CI 1.06–1.60, p<0.05) and syncope 1.58 (95% CI 1.13–2.22, p<0.05).

**Table 2:** Univariate odds ratios (OR) all falls (AF), non- accidental falls (NAF) and syncope in the 12 months prior to wave 1 based on self-reported health variables for TILDA participants (n = 8172).

Conditions	All Falls OR (95% CI)	Non- accidental Falls OR (95%CI)	Syncope OR (95% CI)
**Gender (Female)**	1.02 (0.92–1.14)	1.05 (0.87–1.29)	0.70 (0.57–0.86)
**50–64 years old**	1.02(0.77–1.37)	1.28 (0.73–2.27)	0.93 (0.53–1.62)
**65–75 years old**	1.10 (0.82–1.48)	1.24 (0.69–2.24)	1.17 (0.66–2.07)
**Over 75 years old**	1.07 (0.78–1.45)	1.27 (0.69–2.32)	1.23 (02.68–2.21)
**Anti- hypertensive medications**^1^	1.10 (0.99–1.24)	1.57 *(1.30–1.92)	1.42* (1.16–1.75)
**Depressive symptoms**^2^			
**Mild**	REF	REF	REF
**Moderate**	1.45* (1.27–1.67)	2.09* (1.65–2.66)	1.78 (1.37–2.30)
**severe**	1.88 *(1.59–2.22)	3.01* (2.31–3.93)	2.96 (2.25–3.90)
**Composite CVD**^3^			
**0–2**	1.54* (1.14–2.07)	1.33* (1.02–1.73)	0.99 (.87–1.14)
**3 to 4**	3.59* (2.36–5.46)	1.83* (1.17–2.87)	1.35* (1.05–1.73)
**4 to 5**	1.69 (0.51–5.58)	3.78* (1.73–8.25)	2.24* (1.30–3.85)
**Unsteadiness**^4^	2.74* (2.36–3.19)	4.73* (3.79–5.89)	2.22* (1.70–2.91)
**Self- reported cardiac conditions**			
**Hypertension**	1.08 (0.96–1.29)	1.50*(1.23–1.83)	1.48*(1.20–1.82)
**Angina**	1.41*(1.13–1.75)	1.97*(1.40–2.77)	2.22*(1.58–3.13)
**Heart attack**	0.98 (0.75–1.27	1.2 (0.77–1.87)	1.78*(1.19–2.65)
**Heart failure**	1.64* (1.03–2.62)	2.47* (1.27–4.81)	2.15*(1.03–4.49)
**Stroke**	1.87*(1.29–2.71)	3.50*(2.15–5.69)	3.94*(2.42–6.41)
**Diabetes**	1.36*(1.12–1.64)	1.40*(1.01–1.94)	1.48*(1.06–2.08)
**TIA**	1.82* (1.31–2.52)	2.68*(1.68–4.28)	2.35*(1.41–3.92)
**High Cholesterol**	1.11*(1.00–1.24)	1.21 (0.99–1.48)	1.20 (0.98–1.49)
**Heart murmur**	1.56*(1.25–1.96)	1.45 (0.98–2.16)	1.98*(1.37–2.88)
**Arrhythmia**	1.49*(1.23–1.81)	1.42*(1.01–1.99)	2.05*(1.51–2.81)

^1^Antihypertensive medications (coded by the Anatomic Therapeutic Chemical (ATC) anti-hypertensive medication with ATC code C02, C03, C07, C08, C09)

^2^Depressive symptoms (as measured by Centre for Epidemiological Studies scale (CES-D) scale with scores of < 16 indicating insignificant symptoms for depression; >16 and < 26 indicating moderate to severe depressive symptoms and > 26 indicating severe depressive symptoms.)

^3^composite number of self- reported doctor diagnosed cardiac conditions including angina, hypertension, diabetes, stroke, TIA(transient ischemic attack), heart murmur, high cholesterol and cardiac arrhythmia.

^4^ Self-reported unsteadiness during walking

*Denotes statistical significance at p<0.05

**Table 3:** Adjusted odds ratios † (OR) with confidence intervals (CI) for all falls (AF), non-accidental falls (NAF) and syncope in the 12 months prior to wave 1 (n = 8172).

Conditions	All falls OR (95% CI)	Non-accidental Falls OR (95%CI)	Syncope OR (95% CI)
**Hypertension**	0.96 (0.81–1.12)	1.10 (0.83–1.45)	1.29 (0.97–1.72)
**Angina**	1.11 (0.87–1.42)	1.15 (0.79–1.68)	1.58* (1.08–2.31)
**Heart attack**	0.84(0.64–1.11)	0.81 (0.51–1.29)	1.35 (0.88–2.07)
**Heart failure**	1.26 (0.78–2.06)	1.38 (0.68–2.81)	1.44 (0.67–3.08)
**Stroke**	1.22 (0.81–1.82)	1.65 (0.96–2.85)	2.88*(1.69–4.89)
**Diabetes**	1.21 (0.99–1.48)	0.98 (0.69–1.40)	1.25 (0.88–1.79)
**TIA (transient ischemic attack)**	1.36 (0.96–1.92)	1.55(0.94–2.55)	1.80* (1.05–3.07)
**High Cholesterol**	1.08 (0.96–1.21)	1.10 (0.89–1.36)	1.10 (0.89–1.38)
**Heart murmur**	1.35*(1.07–1.71)	1.09 (0.72–1.65)	1.71*(1.17–2.52)
**Arrhythmia**	1.30*(1.06–1.60)	0.94 (0.65–1.34)	1.58* (1.13–2.22)

^†^Based on logistic regression controlling for age, sex, antihypertensives (coded by the Anatomic Therapeutic Chemical (ATC) anti-hypertensive medication with ATC code C02, C03, C07, C08, C09), depressive symptoms (as measured by Centre for Epidemiological Studies scale with scores of < 16 indicating insignificant symptoms for depression; >16 and < 26 indicating moderate to severe depressive symptoms and > 26 indicating severe depressive symptoms.) and Self-reported unsteadiness during walking

*denotes statistical significance at p<0.05

Table 4 presents the results of multivariate analysis using a composite number of CV conditions. Participants with at least 5 cardiovascular conditions were more likely to report all falls (OR = 2.07, 95% CI 1.18–3.64, p<0.05) and NAF (OR = 2.89, 95%CI 1.28–6.52, p<0.05). Having three to four cardiovascular conditions was associated with increased odds of reporting syncope (OR = 2.62, 95% CI 1.65–4.17, p<0.05). Moderate and severe depressive symptoms were associated with a greater likelihood of reporting any falls, NAF and syncope in the past year (Table 4). Self-reported gait instability showed a statistically significant association with all three conditions as well (all falls OR = 2.49, 95% CI 2.13–2.94, NAF OR = 3.78 95% CI 2.97–4.80, syncope OR 1.50 95% CI 1.12–2.01).

**Table 4:** Multi-variate analysis with odds ratios (OR) of participants reporting all falls (n = 1,579), non-accidental falls (NAF) (n = 406) and syncope (n = 363) in wave one of TILDA (n = 8,172).

Conditions	All Falls OR (95% CI)†	Non-Accidental Falls OR (95% CI)†	Syncope OR (95% CI)†
**Gender (female)**	1.03 (0.93–1.16)	1.05 (0.85–1.29)	0.67 (0.54–0.83)
**AGE**			
**50–64 years old**	0.98 (0.73–1.34)	1.38 (0.76–2.50)	0.76 (0.43–1.36)
**65–75 years old**	1.09 (0.80–1.49)	1.30 (0.71–2.38)	0.98 (0.55–1.75)
**Over 75 years old**	1.08 (0.78–1.49)	1.29 (0.69–2.40)	1.04 (0.57–1.89)
**Anti-hypertensive medication**^1^	0.97 (0.85–1.10)	1.22 (0.97–1.52)	1.12 (0.89–1.42)
**Depression**^2^			
**Mild**	Ref	Ref	Ref
**Moderate**	1.35*(1.18–1.56)	1.86*(1.46–2.36)	1.70* (1.31–2.20)
**Severe**	1.54*(1.29–1.83)	2.17*(1.63–2.87)	2.54* (1.91–3.39)
**Composite CVD**^3^			
**0–2**	0.92 (0.78–1.07)	1.18 (0.87–1.59)	1.22 (0.88–1.71)
**3 to 4**	1.09 (0.82–1.44)	0.99 (0.60–1.62)	2.62*(1.65–4.17)
**4 to 5**	1.91*(1.07–3.39)	2.51*(1.08–5.79)	1.43 (0.42–4.83)
**Unsteadiness**^4^	2.49*(2.13–2.94)	3.78*(2.97–4.80)	1.50*(1.12–2.01)

^†^ Adjusted for each parameter listed in the model (gender, age group category, depression severity, composite cardiovascular disease (CVD) score and unsteadiness during walking).

^1^As coded by the World Health Organisation Anatomic Therapeutic Chemical (ATC) antihypertensive medication with ATC code C02, C03, C07, C08, C09.

^2^As measured by Centre for Epidemiological Studies Depression Scale (CES-D); scores of < 16 indicated insignificant symptoms for depression; >16 and < 26 indicated moderate to severe depressive symptoms and > 26 indicated severe depressive symptoms.

^3^ composite number of self- reported doctor diagnosed cardiac conditions including angina, hypertension, diabetes, stroke, TIA(transient ischemic attack), heart murmur, high cholesterol and cardiac arrhythmia.

^4^ Self-reported unsteadiness during walking

CI = confidence interval

*denotes statistical significance with p-value <0.05

## Discussion

This paper describes the prevalence of all falls (19.2%), NAF (5.1%) and syncope (4.4%) in the past year in a community-dwelling population aged 50 years and older. The prevalence and incidence of all falls and NAF increases with age but the same pattern was not observed for syncope. There are increased odds of reporting all three conditions with increasing number of self-reported cardiovascular conditions.

We have reported a consistent prevalence and incidence rate of all falls of 19.2%. Other community-based studies have reported higher accidental falls rates of 25–30% when measured retrospectively and 35–40% when measured prospectively [21–26]. The younger average age profile in the first wave of TILDA may account for the lower reported yearly prevalence. Consistent with previous studies, the over 75-year age group represents over 20% of falls reported.

Non-accidental falls (NAF), defined as a fall without any obvious slip or trip accounted for about one quarter of all falls, with those over the age of 75 years twice as likely to report NAF as adults aged 50–64 years (8% versus 4%). This study remains the largest community-based cohort to report on the prevalence of NAF and is consistent with a previously reported prevalence of 5% in community-dwelling older adults in New Zealand. [26–28]. In contrast, between 20–50% of all falls presenting to emergency departments are non-accidental[27], perhaps indicating a high morbidity associated with NAF.

We have been able to show unique prevalence and incidence estimates for syncope in the same population. Our cohort has a lower prevalence of syncope to that reported by the Olmstead community cohort (which also focused on older adults), who reported 16.9% overall [29]. TILDA has demonstrated a consistent rate of syncope occurrence at 4 per 1000 person years in our cohort. The Framingham cohort studies had reported higher cumulative incidence rate of 5.7 per 1000 person years in men aged 60–69 years and had a sharp rise to 11.1% per 1000 person years in men aged 70 years and older[30]. They used a definition of syncope that included transient ischaemic attack, stroke and seizures making it difficult to make direct comparison to our cohort. Although at wave two, we reported an increase in syncope incidence in the over 75 age group, it does not demonstrate the same degree of change as reported in the Framingham cohort. This lack of age variation is also in contrast to all falls and NAF and may represent an under-reporting of syncope in older age groups; or the presentation of syncope as a fall.

Low blood pressure, intermittent arrhythmia and heart failure have all shown associations with falls risk in epidemiological studies[31]. Observational studies also support the link between cardiovascular disease and NAF with higher rates of cardiac arrhythmia and carotid sinus syndrome in participants who report NAF[32, 33]. We have demonstrated that increasing cardiovascular co-morbidity were associated with an increase in reporting NAF adding to the evidence linking cardiovascular disease to NAF. Cardiovascular assessment has been enshrined in the original American Geriatrics Society/British Geriatrics Society guidelines for falls prevention and our data would suggest a continued emphasis on the use of a structured cardiovascular assessment as part of a falls prevention work-up[34]. Given the similarities between NAF and syncope, it is recommended that NAF are managed in the same way as syncope in order to realise beneficial responses to intervention [35, 36].

### Strengths and limitations

TILDA provides an opportunity to distinguish between and characterise all falls, NAF and syncope in a large, community-dwelling cohort. Few studies present these together despite the strong overlap between all three. However, there are also some limitations, including the use of self-reported falls and syncope over the past year which relies on a participant’s ability to recall past events leading to recall bias. This may lead to inaccurate reporting of these events when compared to cohorts in which falls are recorded prospectively, for example with falls diaries. Currently, Ireland does not have a unique health identifier which would allow this data to be linked to medical records and therefore, we are unable to clinically verify the final diagnosis and as a result they may suffer from mis-classification bias. Despite this limitation, the cohort is well characterised and will continue to be followed at regular intervals providing a rich source of information as to the exact incidence and associations between falls and syncope in older adults. This cohort was a community dwelling, cognitively intact cohort so results may not pertain to more frail, institutional dwelling older adults.

## Conclusions

We have shown that the prevalence of all falls and NAF is 19.2% and 5.1% respectively in the community-dwelling middle-aged and older population in Ireland. Prevalence of all falls and non-accidental falls in particular are higher in the older age groups. Syncope has yearly occurrence rates of 4.4% and a less consistent age gradient. We have demonstrated that all falls, NAF and syncope have common associations; particularly with increasing cardio-vascular co-morbidity, depressive symptoms and unsteadiness during walking. TILDA represents the largest community-dwelling cohort to present data on all falls, NAF and syncope and allows researchers to focus efforts on untangling the associations between these conditions in order to focus appropriate clinical management strategies and future prevention.
